# Enzymatic activity of PBP1B is required for growth rate-independent ppGpp-mediated resistance to PBP2 inhibitors in *E. coli*

**DOI:** 10.1128/jb.00242-25

**Published:** 2025-12-08

**Authors:** Sarah E. Anderson, Isabella E. Mack, Petra Anne Levin

**Affiliations:** 1 Biology Department, William & Mary 402696, Williamsburg, Virginia, USA; 2 Department of Biology, Washington University in St. Louis 123752, St. Louis, Missouri, USA; University of Florida, Gainesville, Florida, USA

**Keywords:** penicillin-binding protein, beta-lactams, ppGpp

## Abstract

The alarmone (p)ppGpp (ppGpp) accumulates in response to starvation and other stress, leading to inhibition of multiple biosynthetic pathways and, at high concentrations, suppression of bacterial growth. Growth suppression by ppGpp is implicated in the formation of persister cells, which survive antibiotic challenge only to regrow once the drug is removed. However, there is also evidence that low levels of ppGpp contribute to resistance to certain cell wall-active antibiotics in actively growing cells. To characterize ppGpp’s contribution to antibiotic resistance, we measured MICs of a panel of β-lactams in actively growing *Escherichia coli* cells overexpressing a ppGpp synthase (relA*). Cells engineered to modestly overproduce ppGpp exhibited up to 64-fold increases in resistance to PBP2-targeting β-lactams only, with mecillinam the most dramatically affected. Resistance required the transcription factor DksA and the class A penicillin-binding protein (PBP) PBP1B. PBP1B variants defective for transpeptidase activity, glycosyltransferase activity, or both were incapable of supporting resistance, suggesting the full enzymatic activity of PBP1B is required for resistance. Transcriptomics revealed that ppGpp overproduction leads to increased expression of *lpoB*, which encodes an activator of PBP1B. LpoB was required for mecillinam resistance, with an *lpoB* deletion mutant exhibiting a loss of ppGpp-dependent resistance. An *lpoB* deletion strain expressing an LpoB-bypass variant of PBP1B (mrcB*) exhibited an intermediate level of resistance. Together, these results suggest that ppGpp overproduction and the LpoB-dependent enzymatic activity of PBP1B function synergistically to promote survival in the presence of PBP2 inhibitors.

**IMPORTANCE:**

Antimicrobial resistance is an increasing global health threat, but its underlying molecular mechanisms remain incompletely understood. This work clarifies ppGpp’s role in mediating antibiotic resistance in *Escherichia coli*. Elevated levels of ppGpp caused resistance to β-lactam antibiotics targeting the cell wall synthesis enzyme PBP2. Resistance required transcriptional regulation by ppGpp and enzymatic activity of the cell wall enzyme PBP1B. ppGpp overproduction was found to increase expression of the PBP1B activator lpoB. Because ppGpp levels are controlled by nutritional conditions, this work suggests that nutritional availability may impact antibiotic efficacy.

## INTRODUCTION

β-Lactam antibiotics inhibit cell wall synthesis by inactivating penicillin-binding proteins (PBPs). The model bacterium *Escherichia coli* encodes four major biosynthetic PBPs: the class A PBPs PBP1A (*mrcA*) and PBP1B (*mrcB*), which possess both glycosyltransferase and transpeptidase activity, as well as the essential class B transpeptidases PBP2 (*mrdA*) and PBP3 (*ftsI*) (1, 2). The class B enzymes are specialized for different modes of cell wall synthesis; PBP2 is a member of the elongasome that mediates cell elongation, while PBP3 is part of the divisome, required for division (3–5). β-Lactams specific for PBP2 or PBP3 cause cell rounding or filamentation, respectively (5). The roles of the class A PBPs are less clear; PBP1A interacts with both the elongasome and divisome, while PBP1B contributes to division and has been implicated in cell wall repair (6–11). Both enzymes require an outer-membrane activator protein (LpoA for PBP1A and LpoB for PBP1B) for most of their functions (12, 13). Under standard laboratory conditions, the class A PBPs are conditionally essential (14), indicating functional redundancy. However, PBP1A is required for maximal fitness in alkaline conditions, and PBP1B becomes essential in acidic conditions (15), suggesting that these enzymes are also specialized for different environments. These enzymes are required for environment- or mutation-dependent resistance to PBP2- and PBP3-targeting β-lactams (15–17), further supporting a model wherein functional redundancy among the PBPs may allow them to substitute for one another in certain conditions.

The alarmones pppGpp and ppGpp [(p)ppGpp], hereafter referred to collectively as ppGpp, are also major drivers of environmental adaptation in bacteria. In *E. coli,* ppGpp levels are controlled by RelA (a monofunctional ppGpp synthetase) and SpoT (a bifunctional ppGpp synthetase/hydrolase) (18). ppGpp is produced at basal levels during balanced growth; under these conditions, ppGpp is thought to contribute to general homeostatic control, including regulating the balance between longitudinal growth and division (19–21). ppGpp levels vary based on nutrient availability, with levels increasing during poor nutrient conditions. During starvation, ppGpp levels increase up to 100-fold, leading to a cessation of growth; this is known as the stringent response (SR) (19, 22–25).

In *E. coli* and its relatives, ppGpp acts via two regulatory pathways. ppGpp modulates transcription by directly binding to RNA polymerase (RNAP) and the RNAP-binding transcription factor DksA (26, 27), leading to differential expression of hundreds of genes during the SR (28). ppGpp also mediates post-translational regulation by directly binding at least 50 target proteins (25, 29, 30). Although ppGpp’s function in the SR is well understood, its contribution to survival in other stressful conditions, where its levels are lower and growth proceeds more or less normally, is less well characterized.

In addition to aiding survival during starvation, ppGpp also contributes to survival during exposure to antibiotics. Antibiotic tolerance, or the ability of bacteria to survive but not grow during antibiotic exposure, is associated with slowed growth rates (31, 32). ppGpp has been implicated in tolerance to multiple classes of antibiotics (32–36); this is thought to be due to ppGpp’s effects on growth rate, although the molecular mechanism is likely more nuanced (32, 36).

In contrast to tolerance, antibiotic resistance means that bacteria can both survive and grow in the presence of a drug. Mild elevations in ppGpp levels—low enough to reduce, but not completely inhibit, cell growth—are associated with resistance to mecillinam, a β-lactam that targets the essential, elongation-specific class B PBP, PBP2 (37–43). Elevated ppGpp also causes resistance to multiple β-lactams in mutants overexpressing the L,D-transpeptidase gene *ldtD (ycbB)* (44). Overproduction of ppGpp alone does not increase resistance to ampicillin (which targets multiple PBPs), cephalexin (targets the essential, division-specific PBP3), or imipenem (targets multiple PBPs) (44–46), but the effect of ppGpp on resistance to other β-lactams is not known. Both ppGpp-mediated mecillinam resistance and broad-spectrum β-lactam resistance in a ppGpp/LdtD overproducing strain depend on transcriptional regulation by ppGpp (47). Beyond this, the mechanism by which ppGpp causes mecillinam resistance is unclear.

In this work, we systematically evaluated the effects of ppGpp on resistance to β-lactams targeting different PBPs. We found that ppGpp mediates resistance to PBP2-targeting β-lactams only, with mecillinam the most strongly affected. We discovered that ppGpp-mediated mecillinam resistance is dependent on both PBP1B and its activator LpoB. This finding adds to a growing number of examples in which PBP1B makes substantial contributions to mecillinam resistance (15, 17, 43). 

## RESULTS

### Overproduction of ppGpp increases resistance to PBP2-targeting β-lactams

Previous work indicates that mild increases in ppGpp levels lead to mecillinam resistance in *E. coli* (37, 38, 41, 42); however, the effect of ppGpp on resistance to other β-lactams in the absence of mutations remains unclear. To determine whether ppGpp can cause resistance to β-lactams other than mecillinam, we quantified minimum inhibitory concentrations (MICs) for different β-lactams when ppGpp is overproduced. ppGpp overproduction was achieved using a plasmid (prelA*) encoding an IPTG-inducible copy of relA* (*relA_1-455_*), a truncated allele of *relA* encoding only the catalytic domain (48, 49). A plasmid encoding *relA’ (prelA’, relA_1-331_*), a further truncated and inactive allele of *relA*, was used as a negative control (50). Expression was induced with 10 µM IPTG, a concentration that caused only a modest ~25% reduction in growth rate for cells harboring prelA* (Fig. S1A).

**Fig 1:**
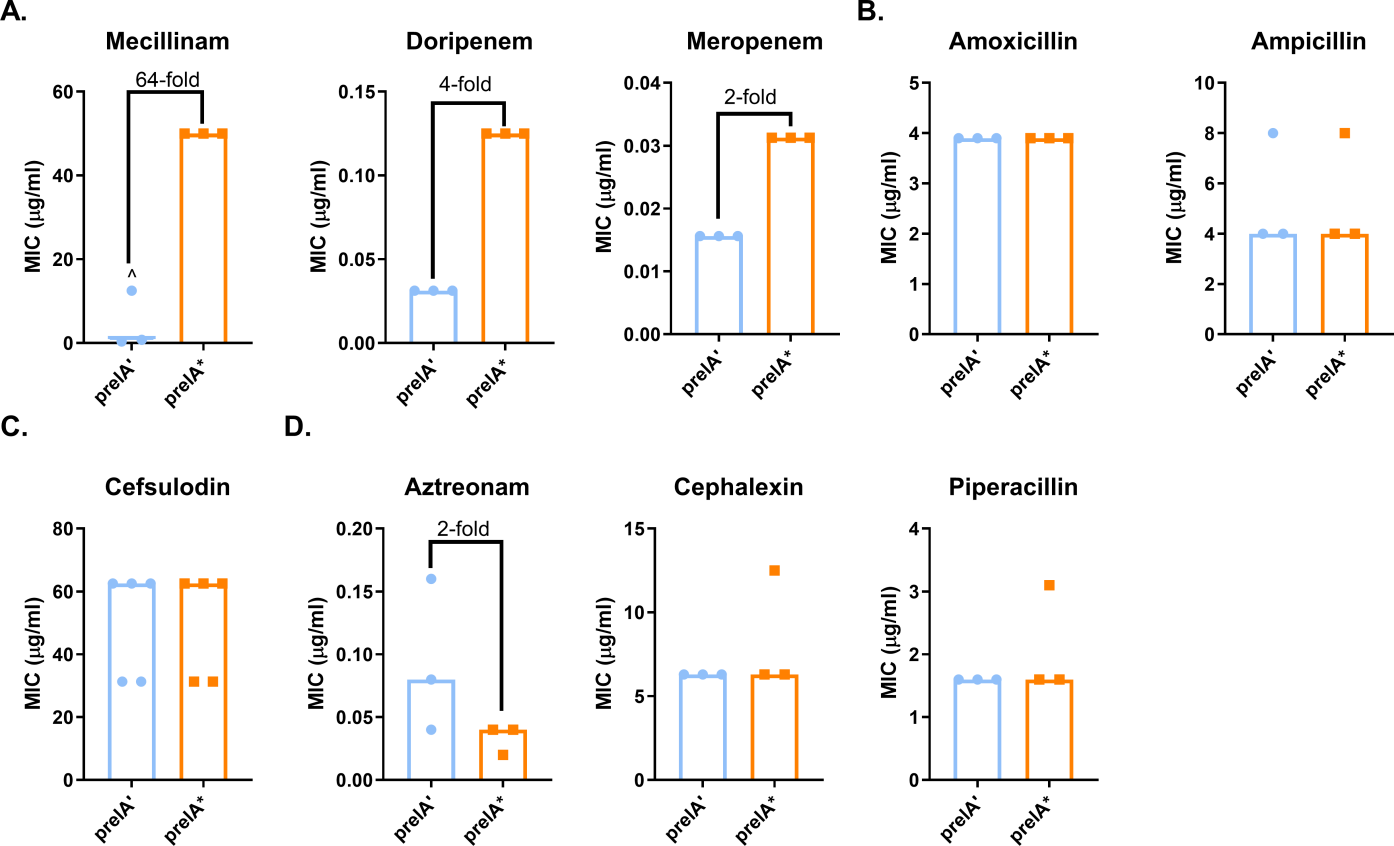
Overexpression of relA* increases resistance to PBP2-targeting β-lactams. (**A**) Modest induction of relA* increases resistance to the PBP2-targeting β-lactams mecillinam, doripenem, and meropenem. (**B–D**) Modest induction of relA* does not increase resistance to non-specific β-lactams (**B**), a PBP1A/PBP1B-targeting β-lactam (**C**), or PBP3-targeting β-lactams (**D**). MICs for at least three biological replicates are shown, along with median MICs and fold changes of median MICs. ^, growth-skipped wells (see Fig. S2A); the next concentration above the highest concentration of drug in which growth was observed was recorded as the MIC.

We measured MICs of a panel of β-lactams that target different PBPs in *E. coli*. Similarly to published results, prelA* caused a 64-fold increase in the median mecillinam MIC (Fig. 1A). prelA* cells grew in the presence of 25 µg/mL mecillinam. For comparison, in previous work, we found that the MIC for MG1655 was 0.4 µg/mL (15).

Intriguingly, the *prelA’* control exhibited an inconsistent response to mecillinam. While two biological replicates had MICs of 1.6 and 0.8 µg/mL, respectively, one replicate grew at 0.4 µg/mL and at 1.6 µg/mL–6.3 µg/mL, but not 0.8 µg/mL (Fig. S2A). We observed similar, but not identical, patterns of inconsistent growth in several subsequent experiments with mecillinam and once with doripenem (Fig. S2). The recurring nature of this inconsistency indicates that it is unlikely to be due to pipetting error and may instead suggest heteroresistance or the accumulation of resistance-associated mutations. This phenomenon was considered beyond the scope of the current study and was not investigated further.

In *E. coli*, mecillinam specifically inhibits PBP2, which is also inactivated by meropenem and doripenem (45, 51). We observed modest but reproducible increases in the median MICs for doripenem (fourfold) and meropenem (twofold) when prelA* was induced (Fig. 1A). We did not observe ppGpp-mediated increases in resistance to β-lactams targeting PBP1A and 1B, PBP3, or multiple PBPs (Fig. 1B through D) (45, 51). Taken together, these results demonstrate that elevations in ppGpp levels increase resistance to PBP2-targeting β-lactams only, with mecillinam being most strongly affected.

To assess the contribution of basal levels of ppGpp to β-lactam susceptibility, we measured MICs for our panel of β-lactams against a strain unable to synthesize ppGpp (ppGpp^0^, Δ*relA spoT::cat*). ppGpp^0^ cells displayed modest ~2-fold reductions in resistance to cefsulodin (targets PBP1A and 1B); doripenem, mecillinam, and meropenem (target PBP2); and piperacillin (targets PBP3). Resistance to aztreonam (targets PBP3) increased twofold, while resistance to the generalists amoxicillin and ampicillin, and the PBP3-targeting cephalexin, was unchanged (Fig. S3). These values suggest that baseline levels of ppGpp are not a major contributor to intrinsic resistance under the conditions tested.

In addition to its reported effects on mecillinam resistance, ppGpp is also associated with β-lactam tolerance (52). To evaluate the impact of modest increases in ppGpp on tolerance, defined as the ability of bacterial cells to survive challenge with super-MIC concentrations of antibiotics, we measured survival of *prelA’* and prelA* cells in inhibitory concentrations of mecillinam, doripenem, cephalexin (PBP3 inhibitor), and ampicillin (non-specific). We found that prelA* reduced killing by all four antibiotics, although the differences for ampicillin were not statistically significant due to high levels of variability between replicates (Fig. S4). This suggests that ppGpp-mediated β-lactam tolerance and resistance occur via different pathways, with resistance occurring to a much more limited subset of antibiotics.

### Reduced growth rate is not sufficient to increase resistance to PBP2-targeting β-lactams

MIC measurements only consider the presence or absence of growth at a defined endpoint (20 h); they do not take into account which strains grew more quickly over the course of the experiment. Lai et al. proposed that ppGpp causes β-lactam resistance through its negative impact on growth rate (43); however, it is unclear why reductions in growth rate would lead specifically to resistance to PBP2-targeting drugs and not β-lactams generally. To determine whether reduced growth rate is sufficient to mediate resistance to these drugs, we compared MICs for wild-type cells grown at 30°C and 37°C. Cells grown at 30°C exhibited a ~30% reduction in growth rate compared to 37°C, similar to the ~25% reduction in growth rate due to overproducing ppGpp (Fig. S1A and B). However, incubation at 30°C did not lead to increased MICs for mecillinam, doripenem, or meropenem. Median mecillinam and meropenem MICs were three- and twofold higher at 37°C than at 30°C, respectively, while median doripenem MICs were unaffected by temperature (Fig. S1C through E). Together, these results demonstrate that modest reductions in growth rate are insufficient to mediate resistance to PBP2-targeting β-lactams, suggesting that ppGpp mediates resistance through other mechanism(s).

### ppGpp-mediated resistance requires DksA

**Fig 2:**
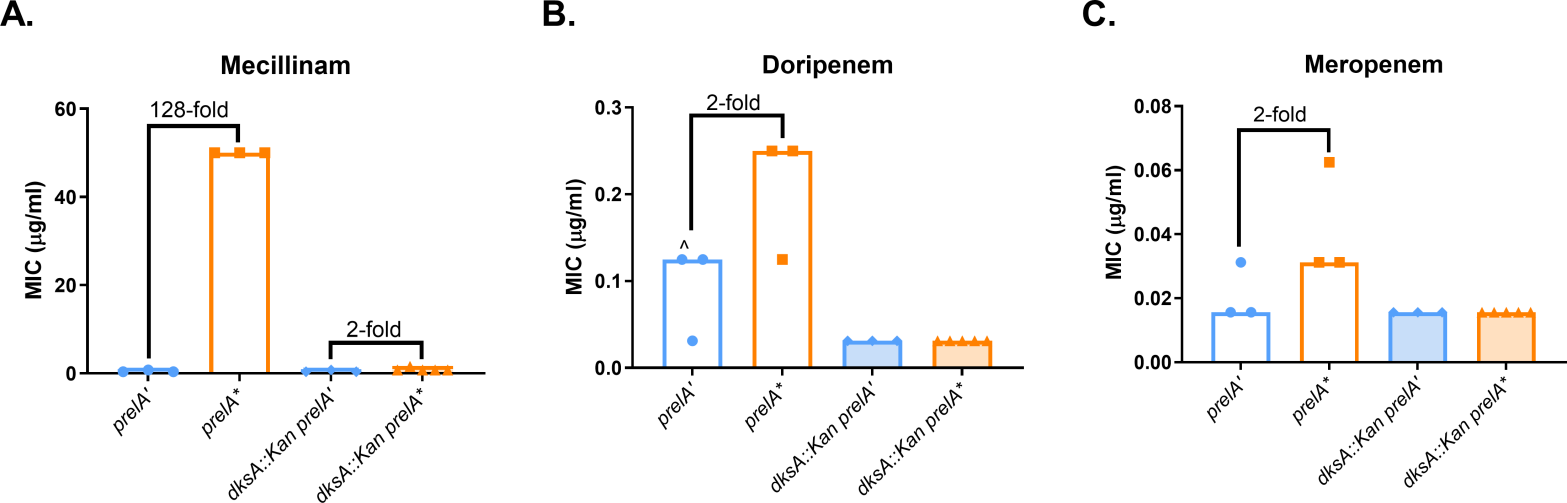
ppGpp-mediated resistance requires *dksA*, suggesting that ppGpp mediates resistance through transcription. Effects of deletion of *dksA* on ppGpp-dependent MICs of mecillinam (**A**), doripenem (**B**), and meropenem (**C**). MIC values for at least three replicates, median MICs, and fold changes of median MICs are shown. ^, growth-skipped wells (see Fig. S2B); the next concentration above the highest concentration of drug in which growth was observed was recorded as the MIC.

ppGpp modulates global biosynthesis via transcriptional and post-translational pathways. Transcriptional regulation by ppGpp occurs in concert with the transcription factor DksA, which facilitates binding of ppGpp to RNAP (26). Recent work suggests that ppGpp mediates mecillinam resistance through RNAP, as mutations in RNAP confer mecillinam resistance in the absence of ppGpp (47). To confirm whether ppGpp’s role in transcription is important for PBP2-targeting β-lactam resistance, we expressed *prelA’* and prelA* in a *dksA::kan* mutant and measured effects on MICs. The *dksA::kan* strain exhibited no ppGpp-dependent resistance to doripenem or meropenem and showed only a twofold increase in mecillinam resistance when prelA* was expressed (Fig. 2). This demonstrates that DksA is required for ppGpp to mediate β-lactam resistance, strongly suggesting that ppGpp mediates resistance through its effects on transcription.

### ppGpp-mediated resistance depends on PBP1B

**Fig 3:**
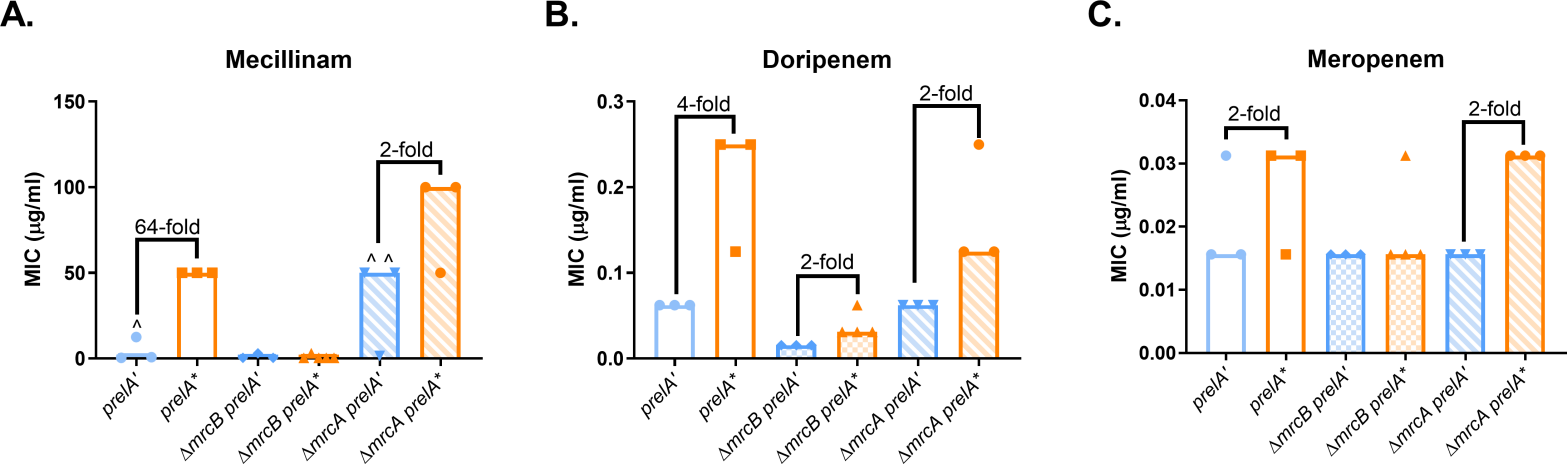
ppGpp-mediated resistance requires PBP1B. Effects of deletion of *mrcB* (encoding PBP1B) and *mrcA* (encoding PBP1A) on ppGpp-dependent MICs of mecillinam (**A**), doripenem (**B**), and meropenem (**C**). MIC values for at least three replicates, median MICs, and fold changes of median MICs are shown. ^, growth-skipped wells (see Fig. S2C); the next concentration above the highest concentration of drug in which growth was observed was recorded as the MIC.

PBP1B also contributes to mecillinam resistance and tolerance (14–17). To determine whether PBP1B or the other major class A PBP, PBP1A, is involved in ppGpp-mediated resistance, we expressed *prelA’* and prelA* in Δ*mrcA* (PBP1A) and Δ*mrcB* (PBP1B) mutants and measured MICs of PBP2-targeting β-lactams. Deletion of *mrcB* fully eliminated ppGpp-mediated mecillinam and meropenem resistance and decreased ppGpp-dependent doripenem resistance (Fig. 3). While prelA* caused a fourfold increase in doripenem resistance in the control strain, this was reduced to a twofold increase in the Δ*mrcB* strain. Interestingly, the Δ*mrcB prelA’* and prelA* strains both exhibited lower doripenem MICs than their respective controls, suggesting that PBP1B also contributes to intrinsic doripenem resistance independently of ppGpp (Fig. 3B). Taken together, these results show that PBP1B is required for ppGpp-mediated β-lactam resistance.

In contrast, PBP1A does not appear to contribute to ppGpp-mediated resistance. The ΔmrcA prelA* strain was highly mecillinam-resistant, with a median MIC of 100 µg/mL (Fig. 3A). We saw high rates of inconsistent growth in mecillinam for the Δ*mrcA prelA’* strain, which elevated the MIC for this strain to 50 µg/mL (Fig. 3A; Fig. S2C). The ΔmrcA prelA* strain exhibited similar meropenem and doripenem MICs to the prelA* control (Fig. 3B and C). Overall, these results suggest that PBP1B, but not PBP1A, is required for ppGpp-mediated resistance to β-lactams, particularly mecillinam.

**Fig 4:**
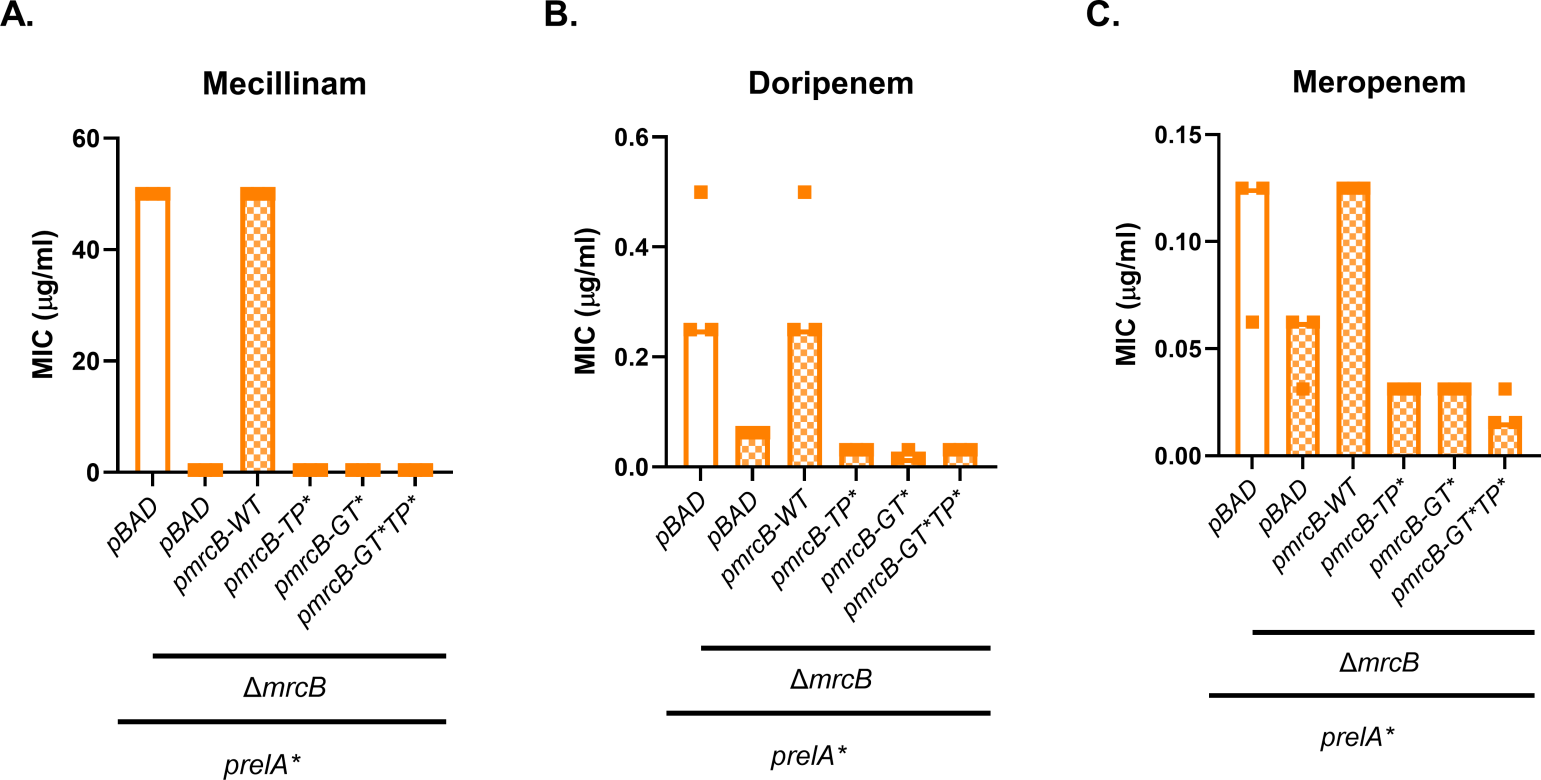
Separation-of-function *mrcB* mutants are incapable of supporting ppGpp-mediated resistance. Effects of disruption of PBP1B transpeptidase activity (TP*), glycosyltransferase activity (GT*), or both enzymatic activities (GT*TP*) on ppGpp-dependent MICs of mecillinam (**A**), doripenem (**B**), and meropenem (**C**). MIC values for at least three replicates and median MICs are shown.

As a bifunctional class A PBP, PBP1B exhibits both transpeptidase (TPase) and glycosyltransferase (GTase) activity. To determine which enzymatic activities of PBP1B are required for resistance, we expressed prelA* in Δ*mrcB* strains expressing separation-of-function alleles of *mrcB* from a plasmid. Mutant variants lacked TPase activity (pmrcB-TP*, *mrcB*_S510A_), GTase activity (pmrcB-GT*, *mrcB*_E233Q_), or both (pmrcB-GT*TP*, *mrcB*_E233Q,S510A_) (53). As expected, deletion of *mrcB* reduced prelA*-mediated mecillinam, doripenem, and meropenem resistance in a strain expressing a pBAD33 control plasmid (Fig. 4). Expression of wild-type *mrcB* from a plasmid (*pmrcB-WT*) fully complemented resistance to all three antibiotics. However, the three PBP1B separation-of-function mutants were unable to restore the ppGpp-dependent resistance phenotype (Fig. 4). It should be noted that disruption of GTase activity in PBP1B dramatically reduces TPase activity (54–56). Therefore, this result demonstrates that the TPase enzymatic function of PBP1B is required for ppGpp-mediated antibiotic resistance and suggests that GTase activity may also contribute.

### ppGpp activates transcription of the PBP1B activator *lpoB*

One explanation for our data so far is that ppGpp could mediate PBP1B activation through DksA-dependent transcription. To determine whether DksA and PBP1B affect resistance through the same pathway, we expressed *prelA’* and prelA* in a Δ*mrcB dksA::kan* double mutant and compared its resistance to each single mutant. Comparing *prelA’* controls for each mutant, the double mutant exhibited a twofold decrease in the median MIC for doripenem and mecillinam compared to the Δ*mrcB* single mutant (Fig. S5), suggesting that there may be a slight additive effect of the two mutations on intrinsic resistance. However, *dksA* and *mrcB* did not exhibit an additive effect on ppGpp-mediated resistance. ppGpp overproduction elicited a twofold increase in mecillinam MIC in each mutant and a twofold increase in doripenem resistance for the Δ*mrcB* and double mutants (Fig. S5A and B). prelA* did not increase meropenem resistance in the double mutant or the *dksA::kan* strain (Fig. S5C). These results suggest that ppGpp likely mediates resistance through a transcriptional pathway involving PBP1B.

To uncover the transcriptional pathway by which ppGpp contributes to β-lactam resistance, we performed RNA-sequencing with *prelA’* and prelA* strains induced with 10 µM IPTG (Data S1). Although many published studies have looked at gene expression changes due to the stringent response, we wanted to determine the effect of chronic, mild overproduction of ppGpp on gene expression. We found over 1,000 differentially regulated genes between prelA* and *prelA’* (Data S1). While we did not find changes in gene expression of *mrcB*, we did observe a statistically significant, ~2-fold increase in expression of *lpoB*, which encodes the PBP1B activator protein (12, 13) (Data S1). Increased *lpoB* expression has also been reported following acute, high-level overproduction of ppGpp (28). A twofold increase in *lpoB* expression has previously been reported to result in a 192-fold increase in mecillinam resistance (17). This raises the possibility that ppGpp may contribute to β-lactam resistance by modestly increasing expression of *lpoB*.

### ppGpp-mediated mecillinam resistance partially requires PBP1B activation by LpoB

**Fig 5:**
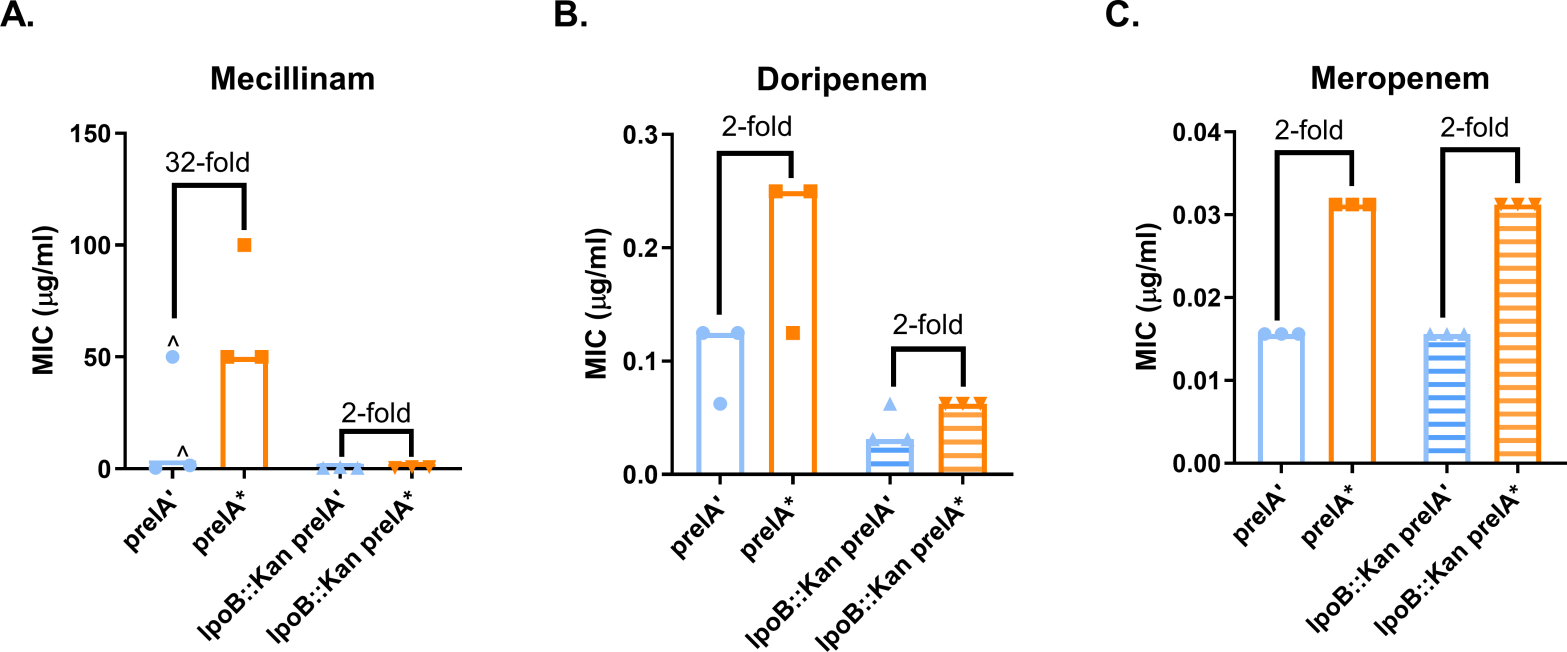
*lpoB* is required for ppGpp-mediated mecillinam resistance. Effects of an *lpoB::kan* deletion on ppGpp-mediated resistance to mecillinam (**A**), doripenem (**B**), and meropenem (**C**). MIC values for at least three replicates, median MICs, and fold changes of median MICs are shown. ^, growth-skipped wells (see Fig. S2D); the next concentration above the highest concentration of drug in which growth was observed was recorded as the MIC.

To assess whether LpoB is required for ppGpp-mediated resistance, we measured the effect of prelA* in an *lpoB::kan* mutant. This mutant exhibited only a twofold increase in the median mecillinam MIC when prelA* was induced, demonstrating that LpoB is required for ppGpp-mediated mecillinam resistance (Fig. 5A). Our results for doripenem and meropenem were less clear. The *lpoB::kan* mutation reduced doripenem resistance in cells expressing *prelA’* and prelA*, although prelA* still caused a twofold increase in resistance in this mutant background (Fig. 5B). Deletion of *lpoB* did not affect the modest twofold increase in meropenem resistance caused by prelA* (Fig. 5C). These results demonstrate that LpoB is required for ppGpp-dependent mecillinam resistance.

As LpoB is typically required for enzymatic activity of PBP1B, it is unclear whether the effect of the *lpoB::kan* mutation on mecillinam resistance is due to a loss of ppGpp signaling to PBP1B through LpoB, or to a general loss of PBP1B activity. To differentiate between these two possibilities, we took advantage of an LpoB-bypass mutant of PBP1B (*mrcB_E313D_*, designated mrcB*) (57). This mutation mimics the effect of LpoB activation on PBP1B, although PBP1B* appears to still be capable of interacting with LpoB (57, 58). We hypothesized that if ppGpp is causing β-lactam resistance by increasing *lpoB* expression, then an mrcB* strain should exhibit ppGpp-dependent resistance, while a mrcB* lpoB::kan mutant should not.

**Fig 6:**
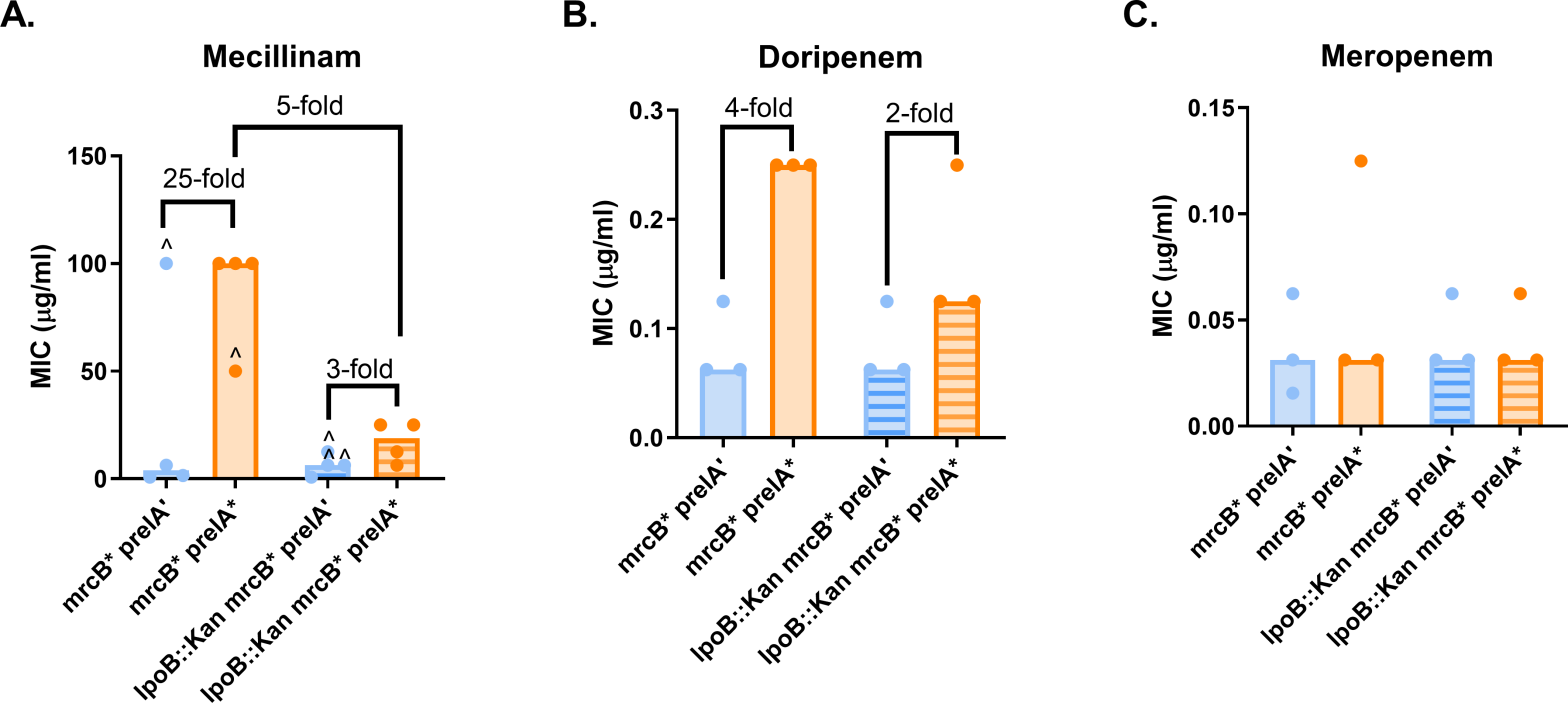
ppGpp mediates mecillinam resistance in part via LpoB. Effect of an *lpoB::kan* mutation on ppGpp-mediated resistance in a strain expressing an LpoB-bypass mrcB* mutation. Resistance was measured to mecillinam (**A**), doripenem (**B**), and meropenem (**C**). MIC values for at least three independent replicates, median MICs, and fold changes of median MICs are shown. ^, growth-skipped wells (see Fig. S2E); the next concentration above the highest concentration of drug in which growth was observed was recorded as the MIC.

Our results suggest that ppGpp-dependent mecillinam resistance is partially dependent on activation by LpoB. The mrcB* mutant exhibited a 25-fold increase in median mecillinam resistance due to prelA*; this was reduced to a threefold increase in the mrcB* lpoB::kan mutant (Fig. 6A). However, the mrcB* lpoB::kan prelA’ control strain exhibited high rates of inconsistent growth at higher mecillinam concentrations (Fig. S2E), inflating the MICs for this strain and contributing to the small fold change due to prelA*. Even so, the median mecillinam MIC for the mrcB* lpoB::kan prelA* strain was fivefold lower than that for the mrcB* prelA* strain. This reduction in MIC suggests that LpoB does contribute to mecillinam resistance even in the mrcB* strain. However, the fact that the mrcB* lpoB::kan strain still responds to ppGpp overproduction suggests that additional, unidentified mechanism(s) also contribute to ppGpp-dependent mecillinam resistance.

LpoB may also contribute to ppGpp-dependent doripenem resistance in the mrcB* strain. We found a fourfold increase in median MIC due to prelA* expression in mrcB* cells; this was reduced to a twofold increase in the lpoB::kan mrcB* strain (Fig. 6B). Surprisingly, the mrcB* and lpoB::kan mrcB* strains both failed to exhibit a reproducible increase in meropenem resistance due to prelA* (Fig. 6C), so the contribution of *lpoB* to ppGpp- and PBP1B-dependent meropenem resistance could not be further explored.

## DISCUSSION

This study demonstrates that ppGpp mediates resistance to a subset of β-lactam antibiotics via a growth rate-independent mechanism. We have confirmed previous findings that ppGpp is responsible for mecillinam resistance and established that ppGpp can cause a mild increase in resistance to doripenem and meropenem (Fig. 1A). These three antibiotics preferentially target PBP2 in *E. coli* (45, 51). This resistance requires the transcription factor DksA (Fig. 2) and catalytic activity by the class A PBP PBP1B (Fig. 3 and 4). Our results indicate that ppGpp stimulates expression of *lpoB*, and that LpoB activation of PBP1B is partially required for resistance (Fig. 5 and 6; Data S1). Together, these data suggest ppGpp may increase resistance in part via LpoB-dependent activation of PBP1B.

This work adds to a growing body of evidence that PBP1B promotes survival when the activity of elongasome-specific components is inhibited. Loss of PBP1B is reported to enhance lysis by both mecillinam and A22, a drug inhibiting the elongasome protein MreB (14, 16, 59, 60), although mecillinam MICs for *mrcB* mutants are similar to wild-type (17, 61). Additionally, defects in PBP1B enhance the lytic phenotype of strains encoding heat-sensitive mutations in elongasome components, including PBP2 (62). Our group identified PBP1B as specifically required for acid-mediated resistance to both PBP2- and PBP3-targeting β-lactams (see below) (15). Multiple studies indicate that cefsulodin (which inhibits both PBP1A and PBP1B) is synergistic with other β-lactams, including mecillinam (63–66), further supporting a protective role for PBP1B during PBP2 inhibition.

While our data indicate that mecillinam resistance is mediated in part via ppGpp-dependent stimulation of PBP1B activity, specifically how PBP1B contributes to resistance remains an open question. PBP1B’s intrinsic TPase activity may substitute—at least in part—for PBP2-mediated transpeptidation. How this substitution would occur is not obvious, as PBP1B is primarily associated with the divisome, while PBP2 is only required for elongation (3, 5, 6, 9–11). One option is that PBP1B contributes to dispersive cell wall synthesis along with other components of the divisome. In support of this idea, modest overexpression of *ftsZ* and other divisome components increases mecillinam resistance and supports growth in PBP2-deficient cells (39, 67). It is also possible that ppGpp-mediated increases in PBP1B’s proposed cell wall repair function mitigate damage due to PBP2 inhibition (6), allowing PBP1B to indirectly compensate for loss of PBP2.

It is formally possible that defects in *mrcB* are masking the beneficial effects of ppGpp overproduction, in line with reports suggesting loss of PBP1B interferes with peptidoglycan integrity (6, 60, 68) and sensitizes cells to a variety of β-lactams (61, 66, 69–71). Although appealing, we feel this model is not supported by our data. Consistent with published results (17, 61), deletion of *mrcB* had no impact on mecillinam and meropenem resistance in cells expressing *prelA’* (Fig. 3). This suggests that loss of PBP1B does not substantially impact sensitivity for either drug in the absence of ppGpp overproduction under the conditions tested. Additionally, our observations that loss of *lpoB* reduces ppGpp-dependent mecillinam resistance in a LpoB-bypass mutant (Fig. 6A), that the effects of *mrcB* and *dksA* on ppGpp-dependent resistance are not additive (Fig. S5), and that ppGpp overproduction increases *lpoB* expression (Data S1) all suggest that ppGpp and PBP1B are operating through a shared pathway.

Significantly, an LpoB-bypass strain lacking *lpoB* reduced, but did not eliminate, ppGpp-dependent mecillinam resistance (Fig. 6A), suggesting that ppGpp also contributes to resistance via additional LpoB-independent pathway(s). Modest increases in ppGpp levels lead to global transcriptional changes (Data S1), including changes in expression of a handful of genes encoding enzymes involved in peptidoglycan synthesis that thus may also contribute to resistance. Our previous work identified a positive relationship between ppGpp levels and the activity (but not the accumulation) of the cell division protein FtsZ, upregulation of which has been implicated in mecillinam resistance (20, 39, 67). While ppGpp has been proposed to mediate β-lactam resistance by reducing expression of rRNA operons, experimental downregulation of rRNA transcription was unable to recapitulate resistance without additional mutations in *rpoB* (47). Importantly, our data argue against the idea that ppGpp mediates PBP2 inhibitor resistance through inhibition of cell growth; reducing growth rate by reducing temperature either reduces MIC (mecillinam and meropenem) or has no impact (doripenem) (Fig. S1).

It is not apparent why ppGpp does not mediate resistance to β-lactams that are not specific to PBP2. ppGpp overproduction can lead to broad-spectrum β-lactam resistance if the L,D-transpeptidase LdtD (YcbB) is simultaneously overexpressed (44, 47). This broad-spectrum resistance requires glycosyltransferase activity from PBP1B, but not transpeptidase activity (44). This is in contrast to the resistance to PBP2-targeting antibiotics observed here, which requires transpeptidase activity of PBP1B (Fig. 4). These findings demonstrate that transpeptidase activity from a non-target enzyme is necessary for β-lactam resistance, but it is not clear why transpeptidase activity from PBP1B is sufficient for resistance to PBP2-targeting antibiotics, while elevated LdtD is needed to confer resistance to other β-lactams. Our findings also seemingly contradict the observation that PBP1B contributes to resistance to both PBP2- and PBP3-targeting antibiotics in low pH conditions (15). It is possible that PBP1B is more highly activated by low pH than by ppGpp, or that this discrepancy is due to additional differences in the cell wall synthesis machinery at low pH versus during ppGpp overproduction.

ppGpp overproduction causes a 64-fold increase in mecillinam resistance, while doripenem and meropenem resistance increase only 2- to 4-fold (Fig. 1A). The low level of doripenem and meropenem resistance due to ppGpp overproduction made it difficult to assess the contribution of LpoB to resistance to these drugs (Fig. 5 and 6). The differences between PBP2-targeting β-lactams may be due to the chemical classes of these drugs, with doripenem and meropenem belonging to the carbapenem class, while mecillinam is a penicillin. All three of these drugs share PBP2 as their specific PBP transpeptidase target (45). However, doripenem and meropenem have also been reported to bind PBP4 (both) and PBP7 (doripenem only) (45), class C PBPs that do not catalyze transpeptidation, and instead modify peptidoglycan in other ways (72). More work is needed to understand why the PBP2-targeting carbapenems are less responsive to overproduction of ppGpp compared with mecillinam.

Finally, this work has implications for the clinical use of mecillinam. While we are not aware of any clinical reports of mecillinam resistance arising solely from mutations that increase ppGpp levels, these mutations are easily isolated in a lab setting (37, 44, 73). Because ppGpp levels vary based on nutritional state, these results also suggest that nutrient-poor environments could cause temporary phenotypic mecillinam resistance, without the accumulation of mutations. Indeed, reversible mecillinam resistance has been observed in wild-type *E. coli* starved for isoleucine and valine (40). Such transient resistance would not be easily detected in a clinical lab setting. Because ppGpp-mediated resistance is entirely dependent on PBP1B, our results suggest that cefsulodin administration may decrease the risk of ppGpp-mediated mecillinam resistance.

## MATERIALS AND METHODS

### Bacterial strains, plasmids, and growth conditions

Bacterial strains, plasmids, and primers used in this study are detailed in Table S1. All strains used were in the MG1655 background, which is referred to as “wild-type.” Selectable mutations were transferred between strains via P1 transduction, with transductants confirmed by PCR (for all mutants) and sequencing (for mrcB* only). The *dksA::kan* mutation was introduced into the Δ*mrcB* background via lambda red recombineering (74). Briefly, the *kan* gene from pKD4 was amplified with primers containing homology to 40 bp flanking *dksA*. The purified PCR product was electroporated into a Δ*mrcB* strain carrying pKD46 induced with 0.7% arabinose (Millipore Sigma); transformants were passaged at 37°C to cure pKD46 and confirmed by PCR.

Plasmids prelA* and *prelA’* were generated from *pALS10* and *pALS14*, respectively, by replacing the *bla* gene with a spectinomycin resistance gene from *pBS58* via *in vivo* assembly (IVA) cloning (50, 75, 76). Plasmids to overexpress separation-of-function *mrcB* alleles were generated by amplifying *mrcB* genes from the plasmids pUM1Bα* (TP*), pUM1BTG*α (GT*), pUM1BTG*α* (TP*GT*), and pUM1Bα (WT) and cloning them into the plasmid *pBAD33* with IVA cloning (53). Primers were obtained from Integrated DNA Technologies (Coralville, IA).

Experiments were performed in LB broth (1% tryptone, 1% NaCl, 0.5% yeast extract). prelA* and *prelA’* plasmids were induced by supplementation with 10 µM IPTG (Gold Bio, St. Louis, MO) and maintained with 100 µg/mL spectinomycin (Gold Bio or Millipore Sigma, St. Louis, MO). *pmrcB* plasmids were induced by addition of 0.2% arabinose and maintained with 30 µg/mL chloramphenicol (VWR, Radnor, PA). Selection for chromosomal mutations was performed using 50 µg/mL kanamycin (Gold Bio or Millipore Sigma) or 12.5 µg/mL tetracycline (Millipore Sigma).

### Minimum inhibitory concentrations

Antibiotics were obtained from Millipore Sigma or Cayman Chemical (Ann Arbor, MI) (doripenem). Cells were isolated from a single colony and grown in LB supplemented with IPTG, spectinomycin, arabinose, and/or chloramphenicol, if appropriate. Cultures were grown at 37°C with shaking until mid-exponential phase (OD_600_ = 0.2–0.6). A 96-well plate was prepared containing twofold serial dilutions of antibiotics in LB supplemented with IPTG, spectinomycin, arabinose, and/or chloramphenicol, as necessary. Cells were inoculated into each well to a final OD_600_ of ~0.0001. Plates were sealed with a Breathe-easy membrane (USA Scientific, Ocala, FL) and incubated at 30°C (Fig. S1C through E) or 37°C (all other experiments) with shaking for 20 h. MICs were determined as the lowest contiguous concentration of drug that prevented visible bacterial growth.

### RNA sequencing

Cultures were inoculated from a single colony and grown in LB supplemented with spectinomycin. Cultures were grown at 37°C with shaking until log phase and back-diluted to an OD_600_ of 0.01 in LB supplemented with spectinomycin and IPTG. Cultures were then grown to log phase and back-diluted again to 0.01 under the same conditions. Once cultures reached an OD_600_ of ~0.2, samples were collected and mixed 8:1 with RNA stop solution (95% ethanol, 5% water-saturated phenol) on ice, followed by centrifugation for 10 min at 4,000 rpm at 4°C. Pellets were flash-frozen in a dry ice-ethanol bath and stored at −80°C. RNA was prepared with the Trizol Plus RNA Purification Kit (Invitrogen, Waltham, MA) supplemented with Max Bacterial Enhancement Reagent (Invitrogen), according to the manufacturer’s instructions. Samples were treated with DNase I (Invitrogen) according to the manufacturer’s instructions. Illumina 12M RNA sequencing with rRNA depletion and fold-change analysis was performed by SeqCenter (Pittsburgh, PA).

### Growth curves

Cultures were grown and diluted into 96-well plates as for MICs, followed by incubation for 20 h in an Epoch 2 plate reader (Fig. S1A) or a Synergy 2 plate reader (Fig. S1B). OD_600_ values were measured every 10 min. Doubling times were calculated from the exponential portion of each growth curve using the Doubling Time Cell Calculator ++ (https://doubling-time.com/compute_more.php). Growth rates (doublings per hour) were calculated by dividing 60 by the doubling time.

### Antibiotic killing curves

Cultures were inoculated from an isolated colony in LB supplemented with IPTG and spectinomycin. Cultures were grown at 37°C with shaking to mid-log phase (OD_600_ = 0.2–0.6) and diluted to an OD_600_ of 0.1. Antibiotic was added to cultures at the indicated concentration, and cultures were incubated at 37°C with shaking. Serial dilutions of cultures were plated on LB supplemented with spectinomycin before antibiotic addition and after 1, 2, 3, 4, 5, 8, and 21 h following addition. CFUs were enumerated following at least 12 h of incubation at 37°C, and percent survival was calculated at each time point compared to t = 0.

### ppGpp^0^ suppressor tests

ppGpp^0^ strains easily accumulate suppressor mutations in RNA polymerase genes (77). For all experiments using ppGpp^0^ strains, cultures were tested for the presence of suppressors at the same time that samples were collected for MICs. Suppressor tests were conducted as previously described (20). Data were only included from experiments where the frequency of suppressors was ≤10%.

### Quantification and statistical analyses

All experiments were performed with at least three biological replicates. Statistical tests were performed as indicated in figure legends using GraphPad Prism 7.

## SUPPLEMENTAL MATERIAL

Data S1 jb.00242-25-s0001.xlsx RNA-sequencing results.

Supplemental material jb.00242-25-s0002.docx Figures S1 to S5, Table S1, and Legend for Data S1.

ASM does not own the copyrights to Supplemental Material that may be linked to, or accessed through, an article. The authors have granted ASM a non-exclusive, world-wide license to publish the Supplemental Material files. Please contact the corresponding author directly for reuse.
