# Supplementary material for: Enzymatic activity of PBP1B is required for growth rate-independent ppGpp-mediated resistance to PBP2 inhibitors in E. coli
Source: J Bacteriol. 2025 Dec 8;208(1):e00242-25. doi: 10.1128/jb.00242-25 (PMC12826052; doi:10.1128/jb.00242-25)
Supplement: Supplemental material — Figures S1 to S5, Table S1, and Legend for Data S1. [file jb.00242-25-s0002.docx]

**Supplemental Figure S1**


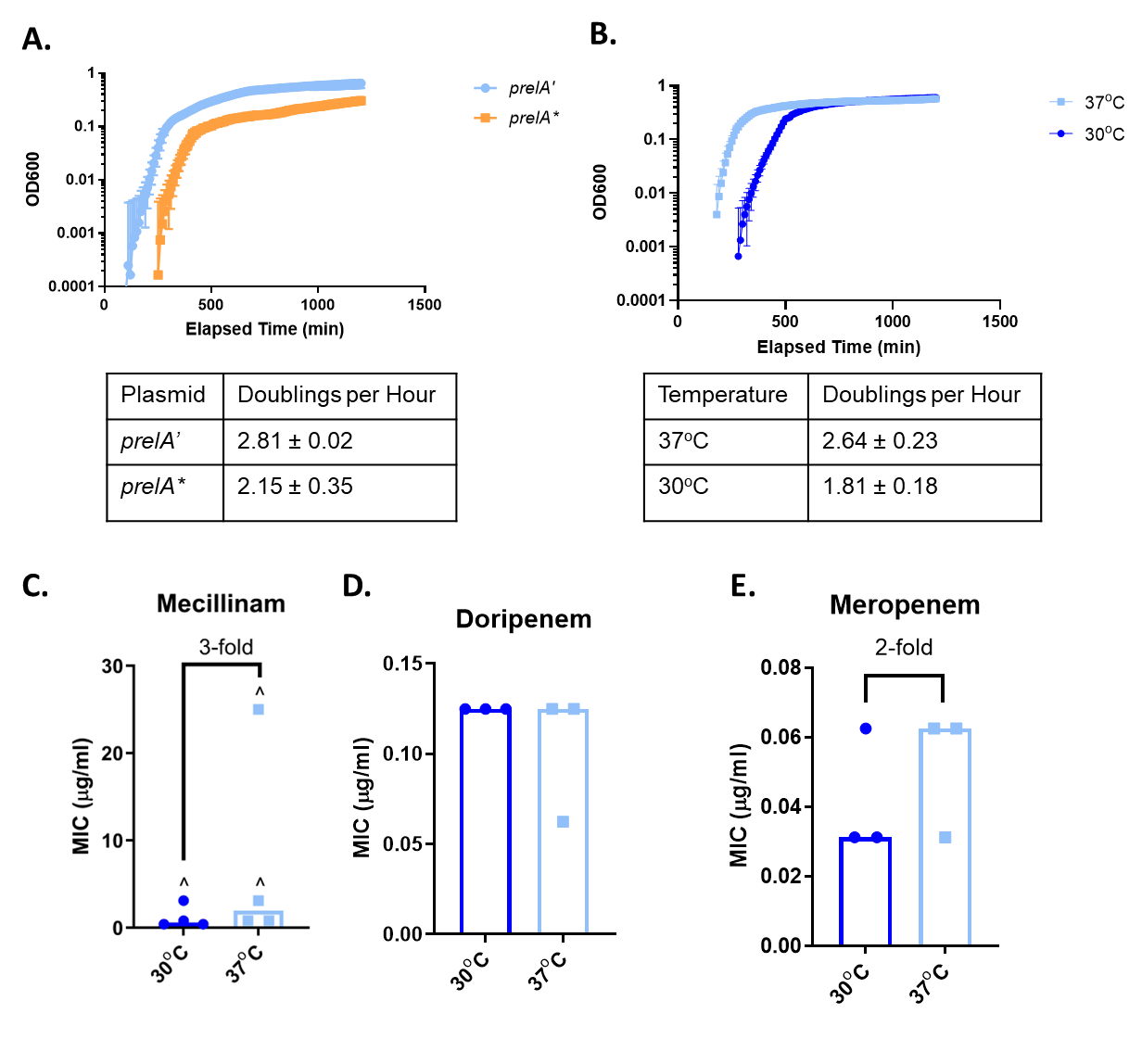


**Supplemental Fig. S1. Decreased growth rate is not sufficient for resistance. A.** Growth curves and growth rates (doublings per hour) of *prelA’* and *prelA** strains induced with 10 µM IPTG in the absence of drug. **B.** Growth curves and growth rates of wild-type cells incubated at 30^o^C or 37^o^C. **C-E.** MICs of wild-type cells incubated at 30^o^C and 37^o^C to mecillinam (**C**), doripenem (**D**), and meropenem (**E**). ^, growth skipped wells; the next concentration above the highest concentration of drug in which growth was observed was recorded as the MIC. Decreased temperature does not mimic the effect of ppGpp production on resistance (**Fig. 1**), suggesting that ppGpp does not cause resistance via lowering growth rate.

**Supplemental Figure S2.**

**A.**

| **[Mecillinam] (µg/ml)** | **100** | **50** | **25** | **12.5** | **6.3** | **3.1** | **1.6** | **0.8** | **0.4** | **0.2** | **0** |
| --- | --- | --- | --- | --- | --- | --- | --- | --- | --- | --- | --- |
| ***prelA’* (1)** |  |  |  |  |  |  |  |  |  |  |  |
| ***prelA’* (2)** |  |  |  |  |  |  |  |  |  |  |  |
| ***prelA’* (3)** |  |  |  |  |  |  |  |  |  |  |  |
| ***prelA** (1)** |  |  |  |  |  |  |  |  |  |  |  |
| ***prelA** (2)** |  |  |  |  |  |  |  |  |  |  |  |
| ***prelA** (3)** |  |  |  |  |  |  |  |  |  |  |  |

**B.**

| **[Doripenem] (µg/ml)** | **2** | **1** | **0.5** | **0.3** | **0.1** | **0.06** | **0.03** | **0.02** | **0.01** | **0.004** | **0** |
| --- | --- | --- | --- | --- | --- | --- | --- | --- | --- | --- | --- |
| ***prelA’* (1)** |  |  |  |  |  |  |  |  |  |  |  |
| ***prelA’* (2)** |  |  |  |  |  |  |  |  |  |  |  |
| ***prelA’* (3)** |  |  |  |  |  |  |  |  |  |  |  |
| ***prelA** (1)** |  |  |  |  |  |  |  |  |  |  |  |
| ***prelA** (2)** |  |  |  |  |  |  |  |  |  |  |  |
| ***prelA** (3)** |  |  |  |  |  |  |  |  |  |  |  |
| ***dksA*::*kan prelA’* (1)** |  |  |  |  |  |  |  |  |  |  |  |
| ***dksA*::*kan prelA’* (2)** |  |  |  |  |  |  |  |  |  |  |  |
| ***dksA*::*kan prelA’* (3)** |  |  |  |  |  |  |  |  |  |  |  |
| ***dksA*::*kan prelA** (1)** |  |  |  |  |  |  |  |  |  |  |  |
| ***dksA*::*kan prelA** (2)** |  |  |  |  |  |  |  |  |  |  |  |
| ***dksA*::*kan prelA** (3)** |  |  |  |  |  |  |  |  |  |  |  |

**C.**

| **[Mecillinam] (µg/ml)** | **100** | **50** | **25** | **12.5** | **6.3** | **3.1** | **1.6** | **0.8** | **0.4** | **0.2** | **0** |
| --- | --- | --- | --- | --- | --- | --- | --- | --- | --- | --- | --- |
| ***prelA’* (1)** |  |  |  |  |  |  |  |  |  |  |  |
| ***prelA’* (2)** |  |  |  |  |  |  |  |  |  |  |  |
| ***prelA’* (3)** |  |  |  |  |  |  |  |  |  |  |  |
| ***prelA** (1)** |  |  |  |  |  |  |  |  |  |  |  |
| ***prelA** (2)** |  |  |  |  |  |  |  |  |  |  |  |
| ***prelA** (3)** |  |  |  |  |  |  |  |  |  |  |  |
| **Δ*mrcA prelA’* (1)** |  |  |  |  |  |  |  |  |  |  |  |
| **Δ*mrcA prelA’* (2)** |  |  |  |  |  |  |  |  |  |  |  |
| **Δ*mrcA prelA’* (3)** |  |  |  |  |  |  |  |  |  |  |  |
| **Δ*mrcA prelA** (1)** |  |  |  |  |  |  |  |  |  |  |  |
| **Δ*mrcA prelA** (2)** |  |  |  |  |  |  |  |  |  |  |  |
| **Δ*mrcA prelA** (3)** |  |  |  |  |  |  |  |  |  |  |  |

**D.**

| **[Mecillinam] (µg/ml)** | **100** | **50** | **25** | **12.5** | **6.3** | **3.1** | **1.6** | **0.8** | **0.4** | **0.2** | **0** |
| --- | --- | --- | --- | --- | --- | --- | --- | --- | --- | --- | --- |
| ***prelA’* (1)** |  |  |  |  |  |  |  |  |  |  |  |
| ***prelA’* (2)** |  |  |  |  |  |  |  |  |  |  |  |
| ***prelA’* (3)** |  |  |  |  |  |  |  |  |  |  |  |
| ***prelA** (1)** |  |  |  |  |  |  |  |  |  |  |  |
| ***prelA** (2)** |  |  |  |  |  |  |  |  |  |  |  |
| ***prelA** (3)** |  |  |  |  |  |  |  |  |  |  |  |
| ***lpoB*::*kan prelA’* (1)** |  |  |  |  |  |  |  |  |  |  |  |
| ***lpoB*::*kan prelA’* (2)** |  |  |  |  |  |  |  |  |  |  |  |
| ***lpoB*::*kan prelA’* (3)** |  |  |  |  |  |  |  |  |  |  |  |
| ***lpoB*::*kan prelA** (1)** |  |  |  |  |  |  |  |  |  |  |  |
| ***lpoB*::*kan prelA** (2)** |  |  |  |  |  |  |  |  |  |  |  |
| ***lpoB*::*kan prelA** (3)** |  |  |  |  |  |  |  |  |  |  |  |

**E.**

| **[Mecillinam] (µg/ml)** | **100** | **50** | **25** | **12.5** | **6.3** | **3.1** | **1.6** | **0.8** | **0.4** | **0.2** | **0** |
| --- | --- | --- | --- | --- | --- | --- | --- | --- | --- | --- | --- |
| ***mrcB* prelA’* (1)** |  |  |  |  |  |  |  |  |  |  |  |
| ***mrcB* prelA’* (2)** |  |  |  |  |  |  |  |  |  |  |  |
| ***mrcB* prelA’* (3)** |  |  |  |  |  |  |  |  |  |  |  |
| ***mrcB* prelA’* (4)** |  |  |  |  |  |  |  |  |  |  |  |
| ***mrcB* prelA** (1)** |  |  |  |  |  |  |  |  |  |  |  |
| ***mrcB* prelA** (2)** |  |  |  |  |  |  |  |  |  |  |  |
| ***mrcB* prelA** (3)** |  |  |  |  |  |  |  |  |  |  |  |
| ***mrcB* prelA** (4)** |  |  |  |  |  |  |  |  |  |  |  |
| ***lpoB::kan* mrcB** prelA’* (1)** |  |  |  |  |  |  |  |  |  |  |  |
| ***lpoB::kan mrcB* prelA’* (2)** |  |  |  |  |  |  |  |  |  |  |  |
| ***lpoB::kan mrcB* prelA’* (3)** |  |  |  |  |  |  |  |  |  |  |  |
| ***lpoB::kan mrcB* prelA’* (4)** |  |  |  |  |  |  |  |  |  |  |  |
| ***lpoB::kan mrcB* prelA** (1)** |  |  |  |  |  |  |  |  |  |  |  |
| ***lpoB::kan mrcB* prelA** (2)** |  |  |  |  |  |  |  |  |  |  |  |
| ***lpoB::kan mrcB* prelA** (3)** |  |  |  |  |  |  |  |  |  |  |  |
| ***lpoB::kan mrcB* prelA** (4)** |  |  |  |  |  |  |  |  |  |  |  |

**Supplemental Figure S2. *prelA’* expressing cells exhibit occasional inconsistent growth and well skipping in mecillinam and doripenem MICs.** Growth patterns for biological replicates used in **Fig. 1A** (**A**), **Fig. 2B** (**B**), **Fig. 3A** (**C**), **Fig. 5A** (**D**), **Fig. 6A** (**E**). Shading indicates visible growth at the indicated concentration of drug; bolded outlines indicate the concentration of drug that was recorded as the MIC.

**Supplemental Figure S3.**

**
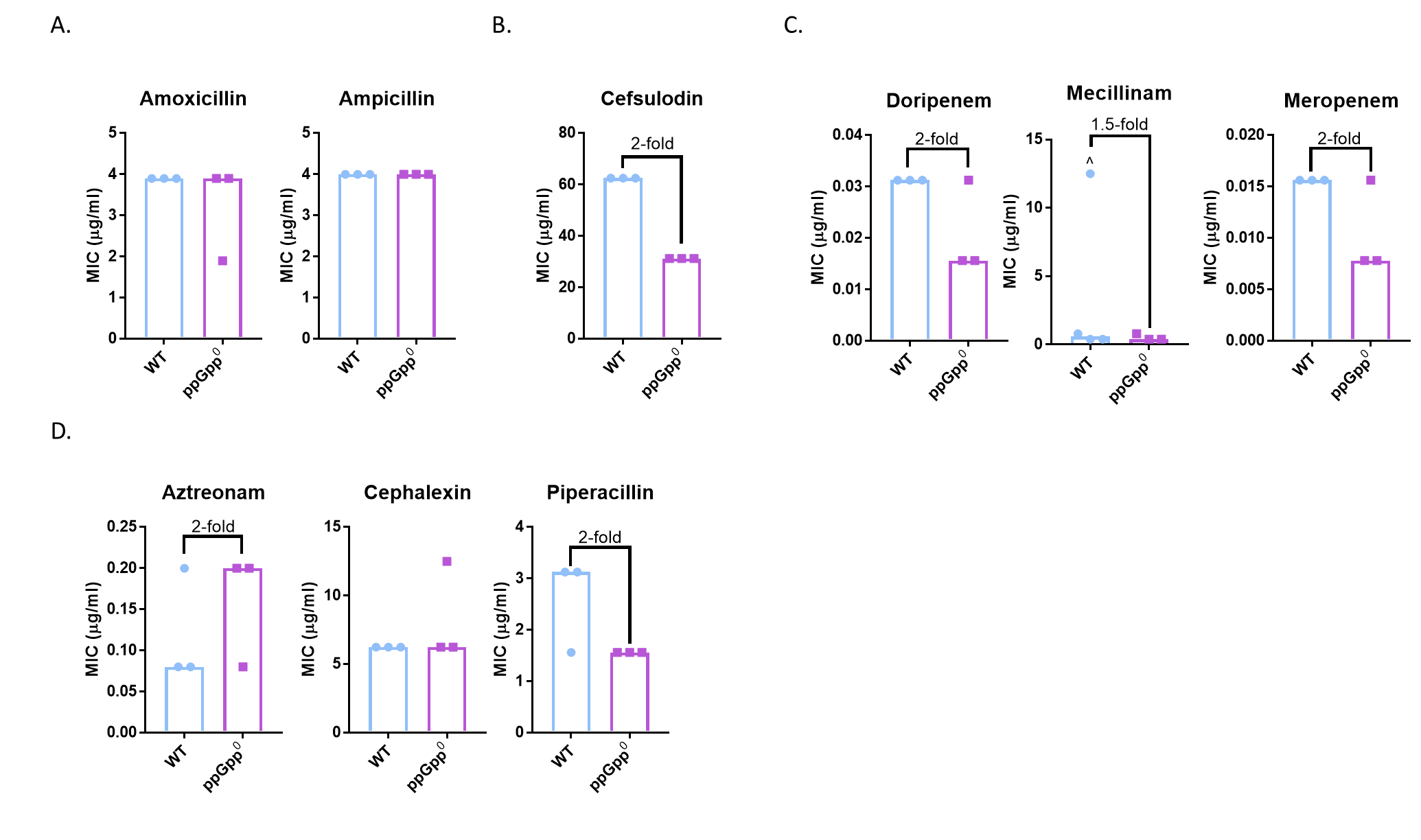
**

**Supplemental Figure S3. Loss of ppGpp (ppGpp^0^) does not greatly impact resistance to beta-lactams.** MICs of ppGpp^0^ cells to β-lactams that are non-specific (**A**), target PBP1A/1B (**B**), target PBP2 (**C**), and target PBP3 (**D**). ^, growth skipped wells; the next concentration above the highest concentration of drug in which growth was observed was recorded as the MIC.

**Supplemental Figure S4.**


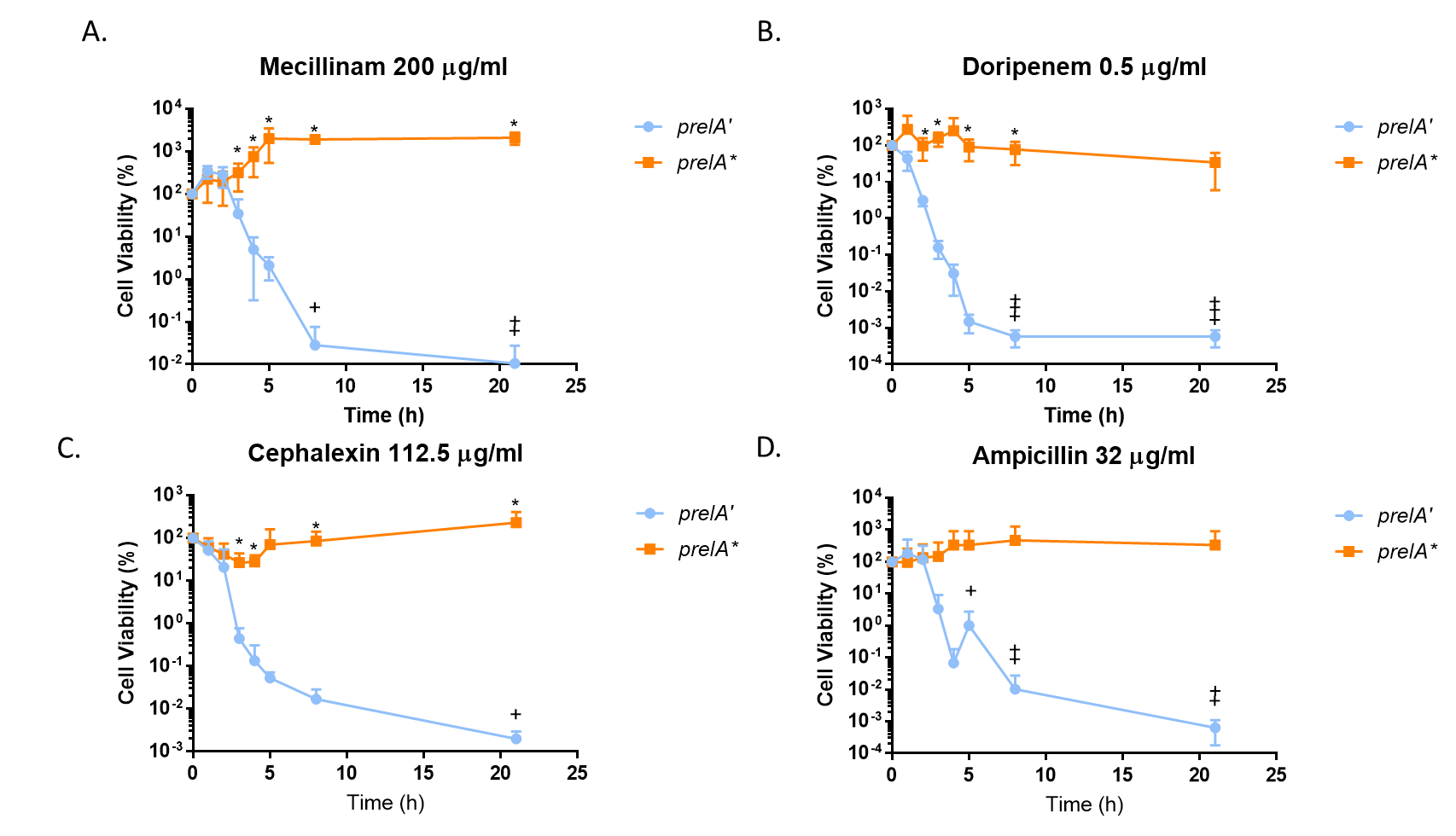


**Supplemental Figure S4. *prelA** decreases killing by β-lactams with different targets.** Survival of strains exposed to inhibitory concentrations of mecillinam (**A**), doripenem (**B**), cephalexin (**C**), and ampicillin (**D**). Data shown represent averages and standard deviations of three independent replicates. *, *p* ≤ 0.05 by two-tailed t-test; +, growth below limit of detection for one replicate; ‡, growth below limit of detection for two replicates;
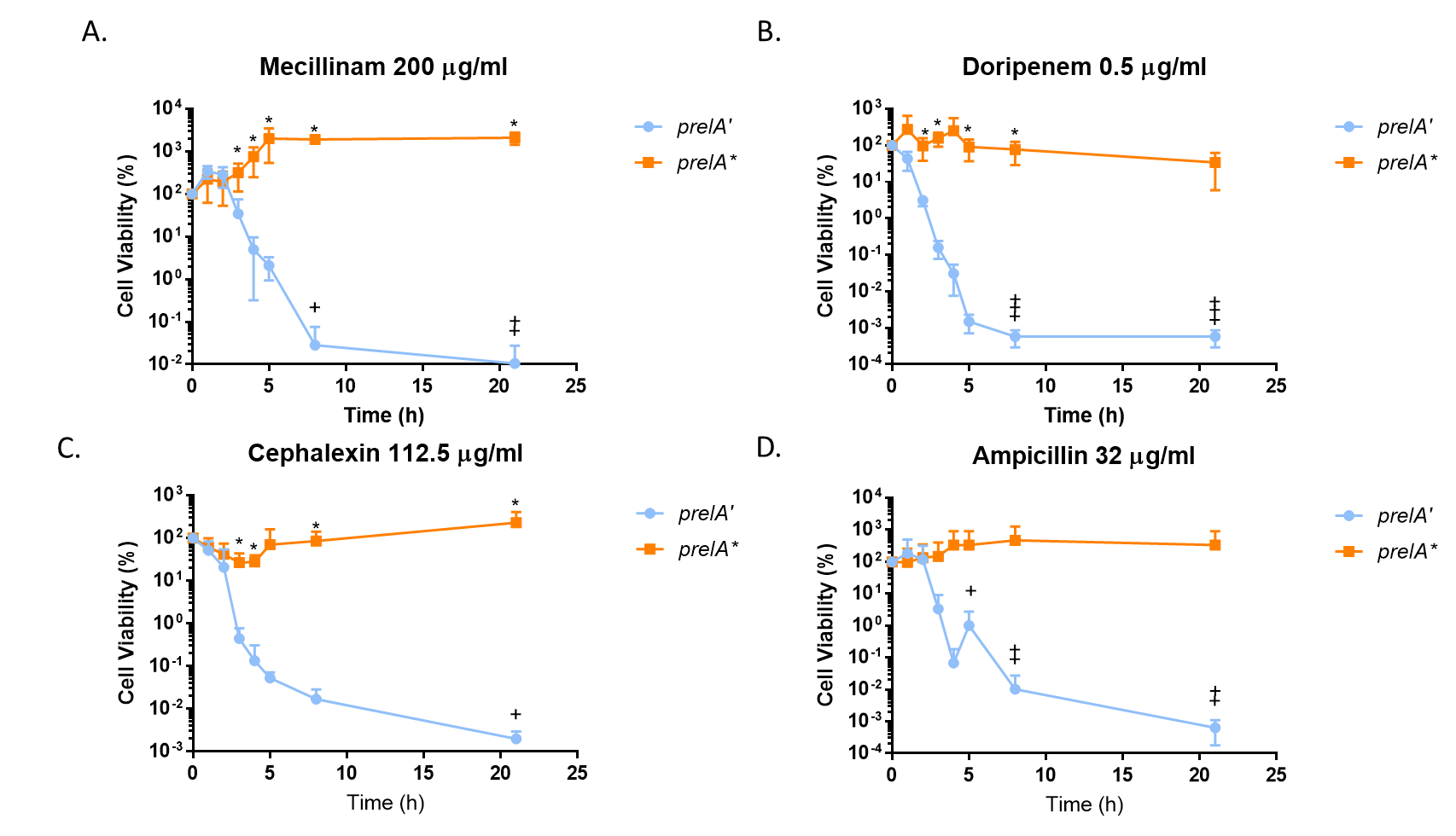
, growth below limit of detection for three replicates. For samples below the limit of detection, the limit of detection was graphed.

**Supplemental Figure S5.**

**
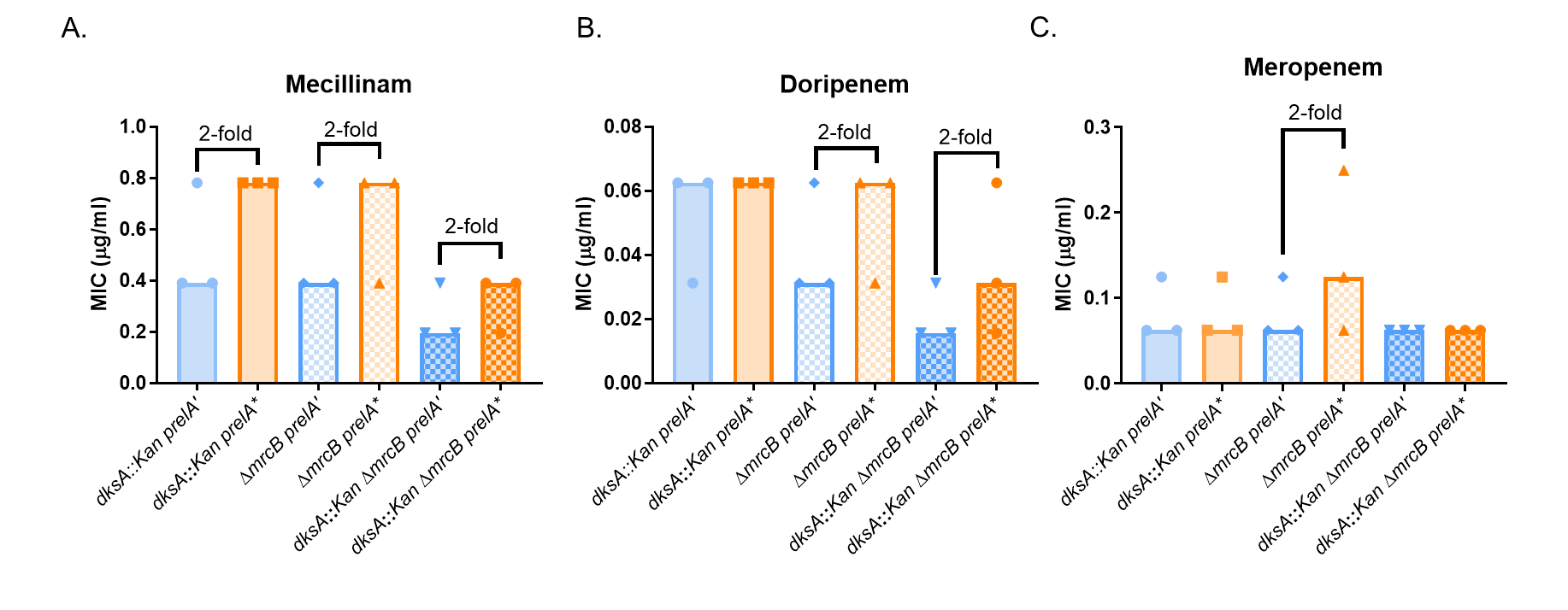
**

**Supplemental Figure S5. Effects of *dksA* and *mrcB* on ppGpp-mediated resistance are not additive.** Effects of deletion of *dksA* and *mrcB* alone and in combination on ppGpp-mediated resistance to mecillinam (**A**), doripenem (**B**), and meropenem (**C**). MIC values for at three replicates, median MICs, and fold changes of median MICs are shown.

**Supplemental Data File S1. Transcriptional differences between cells expressing *prelA’* and *prelA*.*** Cultures were induced with 10 µM IPTG and grown to mid-log phase. Log-2 fold changes (*prelA** vs. *prelA’*), p-values, and individual read values for three biological replicates are shown for all genes and 1,271 differentially expressed genes.

**Supplemental Table S1.** Bacterial strains, plasmids, and primers used in this study.

**Strains**

| **Designation** | **Genotype** | **Source** |
| --- | --- | --- |
| MG1655 (SEA1) | *rph1 ilvG rfb-50 λ- F-* | (1) |
| EAM696 (SEA1116) | MG1655 *mrcB*::*frt* | (2) |
| EAM899 (SEA1117) | MG1655 *mrcA*::*frt* | (2) |
| SEV161 (SEA1137) | MG1655 *dksA*::*kan* | (3) |
| CSW808 (SEA1094) | MG1655 *relA*::*frt* *spoT*::*cat* | (3) |
| SEA1308 | EAM696 *dksA*::*kan* | This study |
| EAM659 (SEA1072) | MG1655 *lpoB*::*kan* | (2) |
| MM43 | MG1655 *mrcB*(E313D) *yadC*::*Tn10* | (4) |
| SEA1069 | MG1655 (SEA1) *mrcB*(E313D) *yadC*::*Tn10* | This study |
| SEA1070 | EAM659 *mrcB*(E313D) *yadC*::*Tn10* | (4) This study |

**Plasmids**

| **Designation** | **Genotype** | **Source** |
| --- | --- | --- |
| *pALS13-spec* (*prelA**) | *lacI^q^* P*_tac_*-*relA*_1-455_  *spc^R^* | This study |
| *pALS14-spec* (*prelA’*) | *lacI^q^* P*_tac_*-*relA*_1-331_ *_­_spc^R^* | This study |
| *pBAD33* | *P_araBAD_ cm^R^* | (5) |
| *pmrcB-WT* | *P_araBAD_-mrcB cm^R^* | This study |
| *pmrcB-TP** | *P_araBAD_-mrcB_S510A_ cm^R^* | This study |
| *pmrcB-GT** | *P_araBAD_-mrcB_E233Q_ cm^R^* | This study |
| *pmrcB-GT*TP** | *P_araBAD_-mrcB_E233Q,S510A_ cm^R^* | This study |
| *pKD4* | *amp^R^kan^R^* | (6) |
| *pKD46* | *P_araBAD_-gam-beta amp^R^* | (6) |

**Primers**

| **Designation** | **Use** | **Sequence** | **Source** |
| --- | --- | --- | --- |
| oSEA211 | To generate *pALS13- and pALS14-spec* | ctgtcagaccaagtttactca | This study |
| oSEA212 |  | agagtttgtagaaacgcaaaa | This study |
| oSEA213 |  | aacttggtctgacaggttagacattatttgccgactac | This study |
| oSEA214 |  | cgtttctacaaactctggcttgttatgactgtttttttg | This study |
| oSEA240 | To generate *pmrcB* plasmids | GCTAGCCCAAAAAAACGGGTA | This study |
| oSEA241 |  | GAATTCGAGCTCGGTACCCG | This study |
| oSEA246 |  | TTTTTTTGGGCTAGCTTTCACACAGGAAACAGAATTC | This study |
| oSEA247 |  | ACCGAGCTCGAATTCCCCGCTTAGATGTTAATTACTACC | This study |
| oSEA306 | To generate SEA1308 | ACATGGGGATCGATAGTGCGTGTTAAGGAGAAGCAACATGgtgtaggctggagctgcttc | This study, adapted from (7) |
| oSEA307 |  | CGTGATGGAACGGCTGTAATTAGCCAGCCATCTGTTTTTCCATATGAATATCCTCCTTAG | This study, adapted from (7) |

**References**

1. Guyer MS, Reed RR, Steitz JA, Low KB. 1981. Identification of a sex-factor-affinity site in E. coli as gamma delta. Cold Spring Harbor symposia on quantitative biology 45 Pt 1:135–40.

2. Mueller EA, Egan AJ, Breukink E, Vollmer W, Levin PA. 2019. Plasticity of Escherichia coli cell wall metabolism promotes fitness and antibiotic resistance across environmental conditions. eLife 8.

3. Anderson SE, Vadia SE, McKelvy J, Levin PA. 2023. The transcription factor DksA exerts opposing effects on cell division depending on the presence of ppGpp. mBio 14:e0242523.

4. Markovski M, Bohrhunter JL, Lupoli TJ, Uehara T, Walker S, Kahne DE, Bernhardt TG. 2016. Cofactor bypass variants reveal a conformational control mechanism governing cell wall polymerase activity. Proceedings of the National Academy of Sciences of the United States of America 113:4788–93.

5. Guzman LM, Belin D, Carson MJ, Beckwith J. 1995. Tight regulation, modulation, and high-level expression by vectors containing the arabinose PBAD promoter. J Bacteriol 177:4121–4130.

6. Datsenko KA, Wanner BL. 2000. One-step inactivation of chromosomal genes in Escherichia coli K-12 using PCR products. Proceedings of the National Academy of Sciences of the United States of America 97:6640–6645.

7. Baba T, Ara T, Hasegawa M, Takai Y, Okumura Y, Baba M, Datsenko KA, Tomita M, Wanner BL, Mori H. 2006. Construction of Escherichia coli K-12 in-frame, single-gene knockout mutants: the Keio collection. Mol Syst Biol 2:2006.0008-2006.0008.
